# Schwannoma of the tongue: a case report with review of literature

**DOI:** 10.1186/s40902-017-0116-2

**Published:** 2017-07-05

**Authors:** Eun-Young Lee, Jae-Jin Kim, Hyun Seok, Ja-Youn Lee

**Affiliations:** Department of Oral and Maxillofacial Surgery, College of Medicine and Medical Research Institute, Chungbuk National University, SeoWon-gu Chungdae-ro 1, Cheong-ju, 28644 South Korea

**Keywords:** Schwannoma, Neurilemmoma, Tongue

## Abstract

**Background:**

Schwannomas (or neurilemmomas) of the tongue are benign, usually solitary, encapsulated masses derived from Schwann cells. Clinical evidence indicates that schwannoma is painless and slow growing. In general, schwannoma is treated by surgical excision.

Here, we describe a case of schwannoma of the tongue, include a review of the literature from 1955 to 2016, and provide data on age, gender, location, presenting symptoms, size, and treatment methods.

**Case presentation:**

A 71-year-old female patient presented with a swelling at the base of the tongue of unknown duration. Magnetic resonance images (MRI) showed a large well-circumscribed solid mass and no significant lymph node enlargement. The mass was excised without removing overlying mucosa.

**Conclusions:**

The authors report a case of lingual schwannoma that was completely removed intraorally without preoperative biopsy. No sign or symptoms of recurrence were observed at 12 months postoperatively.

## Background

Around 25–40% of schwannomas occur in the head and neck region, and of these, 1–12% affect the intraoral area [1], most frequently the tongue or mouth floor [2]. Because of their rarity, intraoral schwannomas are not generally part of the differential diagnosis of tongue mass which includes squamous cell carcinoma, sarcoma, granular cell tumor, salivary gland tumor, schwannoma, leiomyoma, rhabdomyoma, hemangioma, lipoma, lymphangioma, dermoid cysts, and inflammatory lesions [3].

Clinically, schwannomas are benign, usually solitary, encapsulated masses that originate from Schwann cells without pain or ulceration.

Here, we report a case of schwannoma of the tongue base and review the literature. A Google search of the terms “schwannoma (neurilemmoma) of the tongue” and “lingual Schwannoma” was performed from 1955 to 2016. Age, gender, location (anterior, posterior, base, ventral), presenting symptoms, size, and treatment methods were extracted from case reports.

## Case presentation

**Fig. 1:**
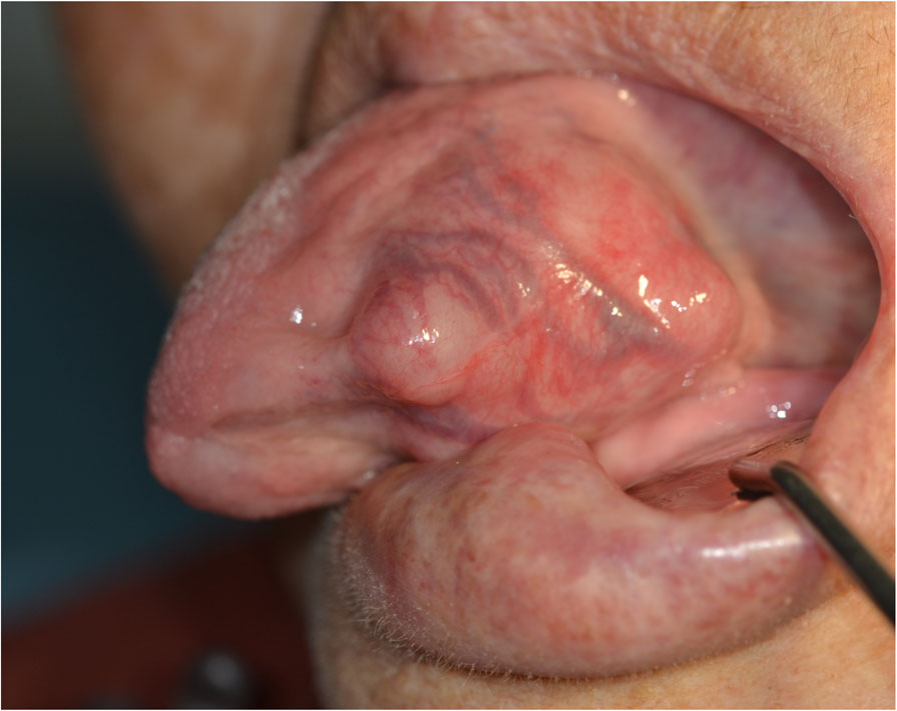
Preoperative intraoral photograph. The mass, which is located in the left tongue base, is covered by normal oral mucosa

**Fig. 2:**
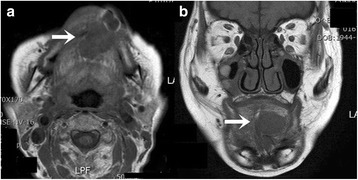
T1-weighted magnetic resonance image showing a well-defined heterogeneous lesion (*white arrow*). **a** Axial view. **b** Coronal view

**Fig. 3:**
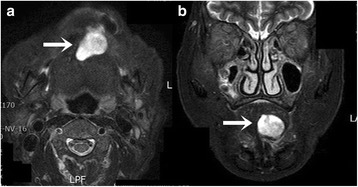
T2-weighted magnetic resonance image showing a well-defined heterogeneous lesion (*white arrow*). **a** Axial view. **b** Coronal view

**Fig. 4:**
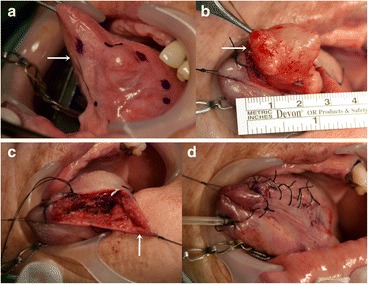
Perioperative clinical photographs. **a** The mass (*white arrow*). **b** The well-encapsulated mass is removed without adhesion (*white arrow*). **c** Photograph of the lesion through an overlying mucosal flap (white arrow). **d** Sutured state

**Fig. 5:**
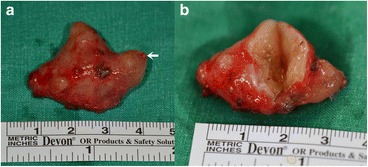
Gross anatomy. **a** Macroscopically, the excised specimen is nodular, soft, and grayish and had dimensions of 2.8 × 2.0 × 3.5 cm. The mass was attached to the lingual nerve (*white arrow*). **b** The cut surface of the mass has a pearly white appearance

**Fig. 6:**
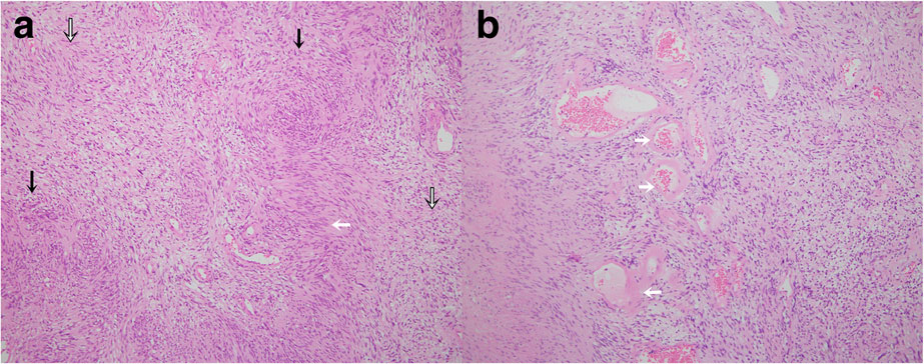
Microscopic examination. The mass is composed of Antoni A (*black arrow*) and Antoni B (*black empty arrow*) regions. **a** Antoni type A consists of closely packed Schwann cells arranged in rows with palisading and elongated nuclei (*white arrow*). **b** Antoni type B of hyalinized vessels in a myxoid background (*white arrow*) (H&E, ×100)

**Fig. 7:**
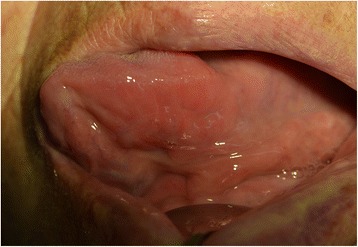
Intraoral photograph obtained at 12 months postoperatively showing no sign of recurrence

A 71-year-old female patient presented with a firm swelling at the base of her tongue of unknown duration that had progressively increased in size. Her only symptom was distortion of the tongue. Medical history taking revealed controlled hypertension (duration *X* years) and thyroid grand tumor. A well-encapsulated nodular mass was evident at physical examination, but without any neurologic symptom or lymphadenopathy in the submandibular area. The mass was 3 × 2 cm sized without ulceration (Fig. 1). Magnetic resonance imaging (MRI) depicted a solid, soft, heterogeneously enhanced lesion (Figs. 2 and 3). Complete surgical excision was conducted under general anesthesia without preoperative biopsy. Blunt dissection was performed without rupturing the mass or causing dehiscence of superficial mucosa. The mass was completely excised under mucosa (Fig. 4). It had been infiltrated by a branch of the lingual nerve, and a portion of the nerve had to be removed to achieve complete resection. On gross examination, the mass was grayish-yellow and well encapsulated with exophytic lobules (Fig. 5). Microscopically, the lesion was characterized by a mixture of Antoni type A and B tissue growth patterns with hyalinized vessel walls (Fig. 6). No sign or symptoms of recurrence were detected 12 months after surgery (Fig. 7).

A review of the literature over the past 61 years that showed 84 cases, including the present case, has been reported (Table 1). Lingual schwannoma may arise at any age between 7 and 77 and shows no sex predilection (44 males and 40 females) [4, 5]. Despite the fact that it originates from nerve tissue, lingual schwannoma is usually painless.

**Table 1:** Patients and tumor characteristics of tongue schwannomas

Author	Year	Gender	Age	Size (cm)	Site	Presentation	Surgical approach
Mercantini and Mopper [21]	1959	M	22	1	Anterior	Intermitten pain	Transoral
Cameron [22]	1959	M	25	1.5	Anterior	Lump	Transoral
Chadwick [23]	1964	F	20	2.2	Posterior	Lump	Transoral
Craig [24]	1964	F	8	3	Posterior	Lump	Transoral
Pantazopoulos [25]	1965	F	45	4.5	Posterior	Dyshagia/change in voice	Transoral
	1965	M	25	1	Anterior	Lump	Transoral
Chhatbar [26]	1965	M	29	5	Posterior	Throat discomfort	Transoral
Firfer et al. [27]	1966	F	28	3	Anterior	Lump	Transoral
Hatziotis and Aspride [28]	1967	M	25	Hazelnut	Posterior	Lump	Transoral
	1967	F	60	Pea	Anterior	Lump	Transoral
Oles and Werthemier [29]	1967	M	52	1	Anterior	Lump	Transoral
Paliwal et al. [30]	1967	M	32	2.5	Anterior	Lump	Transoral
Crawford et al. [31]	1968	M	23	0.5	Anterior	Lump	Transoral
	1968	M	24	1	Anterior	Lump	Transoral
Das Gupta et al. [32]	1969	F	21	5	Posterior	Pain	Transoral
Bitici [33]	1969	M	40	2.5	Anterior	Slight discomfort	Transoral
Sinha and Samuel [34]	1971	M	23	1.5	Posterior	Dysphagia	Transoral
Mosadomi [35]	1975	M	19	3	Anterior	Painful mass	Transoral
Swangsilpa et al. [36]	1976	M	26	3	Anterior	Lump	Transoral
Sharan and Akhtar [37]	1978	F	30	1.5	Anterior	Change in voice	Transoral
Akimoto et al. [38]	1987	M	15	1	Anterior	Lump	Transoral
Sira et al. [39]	1988	F	18	3	Posterior	Lump	Transoral
Flickinger et al. [40]	1989	F	28	3	Anterior	Lump	Transoral
Talmi et al. [41]	1991	F	75	1	Posterior	Lump	Transoral
Gallesio and Berrone [42]	1992	F	21	1.9	Anterior/base	Dysphonia/paresthesia/chewing difficulty	Transoral
Lopez and Ballistin [10]	1993	M	24	0.6	Anterior	Lump	Transoral
Haring [43]	1994	F	49	2	Anterior	Lump	Transoral
Nakayama et al. [44]	1996	F	40	5.5	Anterior	Lump	Transoral
Dreher et al. [15]	1997	F	31	3	Base	Dysphagia	Transoral
Spandow et al. [45]	1999	M	37	7.9	Posterior	Throat discomfort	Transoral
de Bree et al. [2]	2000	F	24	5	Posterolateral/base	Lump	Submandibular
Pfeifle et al. [46]	2001	F	30	0.3	Anterior	Lump	Transoral
	2001	M	18	2	Anterior	Lump	Transoral
Cinar et al. [47]	2004	M	7	1	Anterior	Lump	Transoral
Bassichis and McMlay [48]	2004	M	9	2.3	Posterior/base	Snoring	Transoral
Nakasato et al. [49]	2005	F	9	2	Posterolateral/base	Bleeding/ulceration	Transoral
Hwang et al. [50]	2005	M	23	2.8	Anterior	Lump	Transoral
Lopez-Jornet and Bermejo-Fenoll [51]	2005	M	39	0.8	Posterolateral/base	Lump	Transoral
Vafiadis et al. [52]	2005	M	18	3.1	Anterior	Lump	Transoral
Bansal et al. [53]	2005	M	26	4	Posterolateral/ventral	Paresthesia/dysphonia	Transoral
Hsu et al. [7]	2006	M	20	5	Posterior/base	Bleeding	Transoral
	2006	F	39	4	Posterior/base	Dysphagia	Transoral
	2006	F	32	1.8	Posterior/base	Lump	Transoral
	2006	M	38	3	Anterior	Lump	Transoral
	2006	M	45	0.5	Anterior	Lump	Transoral
	2006	M	25	0.9	Anterior	Lump	Transoral
	2006	F	39	1	Anterior	Lump	Transoral
	2006	M	9	1.2	Anterior	Lump	Transoral
	2006	F	15	1.2	Anterior	Lump	Transoral
	2006	F	12	1.6	Anterior	Lump	Transoral
Ying et al. [54]	2006	F	26	4	Posterior/base	Dysphagia/otalgia	Transoral
Enoz et al. [14]	2006	M	7	2.5	Anterior/base	Dysphagia/pain	Transoral
Mehrzad et al. [55]	2006	M	49	2.2	Posterior/ventral	Pain	CO2-transoral
Batra et al. [56]	2007	M	30	3	Posterolateral/base	Dysphagia, dyspnea, abscess	Transoral
	2007	M	33	3	Posterolateral/base	Dysphonia	Transoral
Ballesteros et al. [57]	2007	F	31	2	Base	Pain	CO_2_-transoral
Sawhney et al. [19]	2008	F	37	4.6	Posterolateral/base	Dysphagia/snoring	Submandibular
Sethi et al. [58]	2008	F	28	1	Anterolateral/ventral	Lump	Transoral
Pereira et al. [59]	2008	M	12	1.5	Posterolateral/ventral	Lump	
Cohen and Wang [17]	2009	M	77	0.7	Posterolateral/ventral	Lump	Transoral
	2009	F	19	1.8	Posterolateral/ventral	Lump	Transoral
Gupta et al. [60]	2009	F	18	1	Anterior/ventral	Lump	Transoral
Mardanpour and Rahbar [61]	2009	M	18	2	Posterior	Dysphagia/change of voice	Transoral
Karaca et al. [62]	2010	F	13	2	Posterolateral/ventral	Dysphagia	Transoral
Cigdem et al. [63]	2010	M	13	2	Anterior/ventral	Lump	Transoral
Jeffcoat et al. [64]	2010	M	68	1.5	Lateral	Lump	Transoral
Naidu and Sinha [65]	2010	M	12	2	Anterolateral/base	Paresthesia/bleeding/ulceration	Transoral
Lukšić et al. [66]	2011	M	10	1.5	Posterolateral/ventral	Lump	Transoral
Batra et al. [67]	2011	F	38	4.2	Posterior/ventral	Dysphagia/change of voice	Transoral
Nisa et al. [68]	2011	F	38	8.5	Posterolateral/ventral	Dysphagia/dysphonia/dyspnea	Transoral
Monga et al. [69]	2013	M	20	2	Posterolateral/base	Lump	Transoral
Lira et al. [5]	2013	F	26	2.5	Posterior/ventral	Cervical pain	Transoral
Erkul et al. [70]	2013	M	21	3	Posterolateral/ventral	Chewing difficulty	Transoral
	2013	M	21	2	Anterolateral/ventral/tip	Lump	Transoral
Jayaraman et al. [71]	2013	F	25	3	Anterolateral/base	Lump	Transoral
George et al. [4]	2014	M	26	4	Posterolateral/base	Dysphagia/dysphonia	Transoral
Bhola et al. [11]	2014	F	14	1.5	Anterolateral/ventral	Lump	Transoral
Moreno-García et al. [16]	2014	F	13	2	Anterior/ventral	Lump	Lip split/mandibulotomy
Nibhoria et al. [72]	2015	F	18	1.5	Posterolateral/ventral	Lump	Transoral
Gopalakrishnan et al. [73]	2016	M	32	3	Posterolateral/ventral	Dysphagia	Transoral
Sharma and Rai [74]	2016	F	20	4	Posterolateral/ventral	Dysphagia/dysphonia	Transoral
Kavčič and Božič [75]	2016	F	20	1.3	Anterolateral/ventral/tip	Lump	Transoral
Lee et al. [76]	2016	M	28	4	Posterior/ventral	Lump	Transoral
Lee	Present case	F	71	3.5	Anterior/base	Lump	Transoral
							Transoral
							Transoral

*MRI* magnetic resonance images, *CT* computed tomography

In 51 cases, the only presenting symptom was an enlarging lump. Other symptoms were dysphagia (15 cases), pain (or discomfort, 10 cases), dysphonia (6 cases), voice change (5 cases), paresthesia (3 cases), snoring (2 cases), bleeding (2 cases), ulceration (2 cases), and abscess (1 case). Masses were located in any part of the tongue. Average size at removal was 2.4 cm (range, 0.3–8.5 cm), and all were treated by transoral excision except 3 cases. The submandibular approach was used in 2 cases and lip splint and mandibulectomy in 1 case. In all three of these cases, masses were located in posterolateral bases.

### Discussion

Although the etiology of schwannoma is not clear, it is known to be derived from nerve sheath Schwann cells, which surround cranial, peripheral, and autonomic nerves [6, 7]. The head and neck are rather common location of this neoplasm. Intraoral schwannomas mainly arise from the tongue, followed by the palate, mouth floor, buccal mucosa, gingiva, lip, and vestibule [8, 9], though the tongue is most commonly involved [10]. The lesion is slow growing, and thus, its onset is usually long before presentation. Lingual schwannoma shows no age or gender predisposition [11]. Usually, it is presented as a painless lump in any part of the tongue of average size 2.4 cm. However, when the mass exceeds 3.0 cm, dysphagia, pain (or discomfort), dysphonia, and voice change are usually presented (Table 1).

Computed tomography (CT) usually shows well-defined homologous lesions. When a heterogeneous lesion is observed by CT, malignant change may be suspected [12]. However, MRI is superior to CT at depicting lingual schwannoma, as it is not degraded by dental artifacts that plague CT in the intraoral area. Lesion signals are isointense versus muscle on T1-weighted images, but hyperintense on T2-weighted images [13]. MRI also allows mass size to be accurately measured and mass localization in relation to other structures. Characteristically, these tumors usually appear to be smooth and well demarcated and do not invade the surrounding structures.

In our case, MRI ruled out the possibility of malignancy and invasion. Enoz et al. [14] reported a malignant transformation rate for head and neck schwannoma of 8–10%. In general, schwannoma does not undergo malignant transformation [15, 16]. However, several cases of malignant transformation of head and neck schwannomas have been reported, although only one involved the tongue [17]. One malignant transformation was evident in our patient.

Histologically, all schwannomas are encapsulated, and beneath capsules, two main patterns are observed, that is, Antoni type A, which is highly cellular and is composed of elongated Schwann cells, which exhibit a palisading nuclear pattern, and Antoni type B, which is also composed of elongated Schwann cells, but cells are arranged in a less dense myxoid manner and are more disorganized than Antoni type A (Fig. 6).

Schwannomas are usually treated by surgical excision with involved originating nerve [18]. In the literature, transoral excision is the most common approach used (Table 1), although some other approaches have been reported to produce success results, such as the submandibular, which is adopted to address lingual schwannoma of the posterolateral base. More recently, CO_2_ laser excision has also been used to treat base of tongue Schwannomas [5, 17]. On the other hand, if a mass is located at the posterolateral base, is inaccessible via the mouth, and has a size >4.0 cm, open techniques, such as the submandibular or lip split approach, are used [2, 4, 19]. Schwannomas are not responsive to radiotherapy [9], and incomplete surgical excision may result in recurrence, although recurrence is uncommon after complete surgical excision [20]. Because masses are encapsulated, their complete removal is straightforward. In our patient, overlying mucosa was preserved to minimize postoperative complications and promote rapid healing without inflammation, and during follow-up, she reported little inconvenience.

## Conclusions

Lingual schwannoma is a relatively rare tumor of the head and neck and may occur anywhere in the tongue. At presentation, the majority of patients complain an asymptomatic mass and slight ulceration. Transoral resection preserving overlying mucosa allowed us to remove the tumor in a manner that precluded recurrence and prevented tongue dysfunction.
